# Stereotactic radiotherapy for metastatic brain tumors: A comparative analysis of dose distributions among VMAT, Helical TomoTherapy, CyberKnife, Gamma Knife, and ZAP‐X

**DOI:** 10.1002/acm2.70046

**Published:** 2025-03-05

**Authors:** Toshihiro Suzuki, Masahide Saito, Ryutaro Nomura, Hikaru Nemoto, Naoto Yanagisawa, Ryuma Sawada, Zennosuke Mochizuki, Naoki Sano, Hiroshi Onishi, Hiroshi Takahashi

**Affiliations:** ^1^ CyberKnife Center Kasugai General Rehabilitation Hospital Yamanashi Japan; ^2^ Department of Radiology University of Yamanashi Yamanashi Japan; ^3^ Department of Neurosurgery Kamiyacho Neurosurgical Clinic Tokyo Japan; ^4^ Division of Radiation Oncology Aizawa Comprehensive Cancer Center Aizawa Hospital Nagano Japan; ^5^ Department of Neurosurgery Kasugai General Rehabilitation Hospital Yamanashi Japan

**Keywords:** CyberKnife, Gamma Knife, Helical TomoTherapy, metastatic brain tumor, VMAT, ZAP‐X

## Abstract

This study evaluates various radiotherapy techniques for treating metastatic brain tumor (BT), focusing on non‐coplanar volumetric modulated arc radiotherapy (NC‐VMAT), coplanar VMAT (C‐VMAT), Helical TomoTherapy (HT), CyberKnife (CK), Gamma Knife (GK), and ZAP‐X. CT images and structures of 12 patients who underwent CK for a single BT were utilized. Twelve treatment plans were created for each planning device. All plans adopted the approach of prescription doses to planning target volume D99.5%. They were divided into stereotactic radiosurgery (SRS) (prescription dose; 21–23 Gy) and stereotactic radiotherapy (SRT) (prescription dose; 30–36.5 Gy) groups and the same parameters evaluated included Gradient Index (GI), Paddick Conformity Index (CI), and treatment time (t‐time). In the SRS group, mean values of GI and CI values were: NC‐VMAT (4.28, 0.60), C‐VMAT (5.61, 0.44), HT (4.68, 0.42), CK (4.31, 0.61), GK (2.81, 0.82), and ZAP‐X (2.99, 0.80). In the SRT group: NC‐VMAT (3.27, 0.84), C‐VMAT (3.81, 0.82), HT (3.76, 0.65), CK (2.98, 0.77), GK (2.61, 0.90), and ZAP‐X (2.80, 0.84). There were no significant differences in the mean values of CI and GI between ZAP‐X and GK in both groups (*p* > 0.05). NC‐VMAT and C‐VMAT had shorter t‐time than other techniques in both groups. ZAP‐X is relatively superior in CI and GI for small tumors, similar to GK, while differences with NC‐VMAT and CK diminish as tumor volume increases. ZAP‐X, CK, and GK have longer t‐time than other treatment techniques, regardless of volume.

## 1 INTRODUCTION

Brain metastases are reported to manifest in approximately 20% to 40% of the entire cohort of cancer patients.^1^, ^2^ Treatment options include surgical intervention, targeted therapy, immunotherapy, and radiation therapy. Furthermore, there are cases where these are combined.^3^ Presently, within the domain of radiotherapy, an array of techniques is available, including volumetric modulated arc radiotherapy (VMAT), stereotactic radiosurgery (SRS), and stereotactic radiotherapy (SRT).^4^ Treatment devices encompass a range of instruments, including linear accelerator (LINAC), Helical TomoTherapy (HT, Accuray, Sunnyvale, CA), CyberKnife (CK, Accuray, Sunnyvale, CA), Gamma Knife (GK, Elekta Instruments AB, Sweden), and ZAP‐X system (ZAP‐X, Zap Surgical Systems Inc, San Carlos, CA). Among these, ZAP‐X is a gyroscopic radiosurgery device developed in recent years for treating intracranial and cervical spine lesions. It has been approved by the regulatory authorities in our country and is in operation in several institutions. ZAP‐X is fully self‐shielded and uses a 3MV LINAC. In addition, it is equipped with a tungsten rotary collimator that offers a selection of eight different collimator sizes ranging from 4 to 25 mm. The 3 MV x‐rays are delivered to the target at a dose rate of 1500 MU/min with a short source‐to‐axis distance of 450‐mm. During irradiation, the actual transmitted dose can be monitored in real‐time by a MV imager positioned diagonally to the LINAC. If a discrepancy greater than 10% is detected compared to the expected transmitted dose, the irradiation is halted.^5^, ^6^ There are a few of comparative studies between ZAP‐X and various treatment devices, likely because ZAP‐X has been relatively recently introduced.^7^, ^8^, ^9^ This study aims to evaluate and compare the dosimetry and irradiation parameters of non‐coplanar VMAT (NC‐VMAT), coplanar VMAT (C‐VMAT), HT, CK, and GK with those of ZAP‐X in treatment planning for metastatic brain tumor (BT).

## 2 METHODS

### 2.1 Patients

This retrospective study was approved by the institutional review board of our hospital (receipt number: 2023–4). From March 2019 to October 2023, 12 patients who underwent BT radiotherapy using CK were enrolled. Informed consent was obtained in the form of opt‐out on the website.

### 2.2 Treatment plans

All Computed Tomography (CT) images were acquired using Optima CT660 (GE Medical Systems, Milwaukee, WI) in the following settings: 120 kV, 400 mA, 1.25‐mm slice thickness, 300‐mm field of view, and 512 × 512 pixels. All structures were delineated with CT and Magnetic resonance images (Vantage Elan 1.5 T, Canon Medical Systems, Japan) on CK MultiPlan Treatment Planning Systems (TPS) (Accuray). CT images and structures were transferred to the respective TPS via DICOM‐RT for treatment planning. Structures included gross tumor volume (GTV), planning target volume (PTV, GTV + 1.0‐mm or 1.25‐mm), brain stem, eye, lens, optic nerve, and optic chiasm. Three clinical medical physicists, one neurosurgeon, one radiological technologist created 72 treatment plans for NC‐VMAT, C‐VMAT, HT, CK, GK, and ZAP‐X using their respective TPS based on the acquired data. The planners responsible for each TPS have more than three years of experience; ZAP‐X, neurosurgeon; VMAT (NC‐VMAT, C‐VMAT), medical physicist; HT, medical physicist; CK, medical physicist; GK, radiologic technologist, respectively. Since each treatment device has slight variations in the technical approach to treatment planning, the plans were designed without constraints on planning time; instead, all plans were designed to bring the prescribed dose as close as possible to 99.5% of the PTV, with a maximum dose within the PTV set at 200% of the prescribed dose. All plans were created based on the organ at risk (OAR) dose constraints of TG 101,^10^ although this study did not include cases with OAR in close proximity. Table 1 shows the characteristics of the 12 patients. Prescription doses and number of fractions were calculated using values used in actual clinical practice. Twelve patients (median [range] age 63.5 [46–91] years; 5 males and 7 females) with a single BT each were included in the study. In this study, to assess differences based on the size of the target, patient 1 to 6 were considered the SRS group (36 treatment plans), while patient 7 to 12 were designated as the SRT group (36 treatment plans) for comparison.

**TABLE 1:** Characteristics of the 12 patients.

Patient	Age	Sex	Primary lesion	Number of Fraction	Prescription dose (Gy)	Target size (cc)	Target localization
SRS group							
1	64	F	Lung cancer	1	23	1.0	R frontal
2	84	F	Lung cancer	1	22	0.7	R parietal
3	66	M	Lung cancer	1	22	0.3	L frontal
4	53	F	Breast cancer	1	22	0.4	R occipital
5	49	F	Lung cancer	1	22	0.4	R temporal
6	58	F	Breast cancer	1	21	0.4	R cerebellum
SRT group							
7	75	F	Colon cancer	3	32.5	6.1	L frontal
8	63	M	Rectum cancer	3	32.5	26.9	R occipital
9	46	M	Melanoma	4	36.5	24.0	L frontal
10	91	M	Melanoma	4	36.5	6.2	R cerebellum
11	58	F	Breast cancer	3	30	2.4	R parietal
12	78	M	Lung cancer	4	32	7.8	L frontal

*Note*: Patients 1 to 6 were considered the stereotactic radiosurgery group, patients 7 to 12 were designated as the stereotactic radiotherapy group. Prescription doses and number of fractions were calculated using values used in actual clinical practice.

### 2.3 NC‐VMAT

For NC‐VMAT, treatment planning was performed by RayStation version 10A (RaySearch Laboratories, Stockholm, Sweden). All plans were created with one coplanar full arc (181°−179° clockwise, couch angle 0°) and three non‐coplanar half arcs. The collimator angles of 15° were used for both the full arc and half arcs. The gantry angles for the three half arcs were 179° to 359° (counterclockwise), 359° to 179° (clockwise), and 181° to 359° (clockwise), with couch angles set at 315°, 270°, and 45°, respectively. The prescription dose was calculated using the collapsed cone convolution algorithm on a 1 × 1 × 1‐mm resolution. All NC‐VMAT plans were delivered by 6 MV FFF x‐ray beams of Elekta Synergy unit with an Agility gantry head, which has 160 MLC leaves of 5‐mm (Elekta AB, Stockholm, Sweden).

### 2.4 C‐VMAT

For C‐VMAT, treatment planning was also performed by RayStation version 10A. All plans used one full arc (181°−179° clockwise) with a collimator angle of 15°, couch angle of 0°. All C‐VMAT plans were also delivered by 6 MV FFF x‐ray beams of Elekta Synergy unit with an Agility gantry head, which has 160 MLC leaves of 5‐mm.

### 2.5 HT

For HT, treatment planning was performed by TomoTherapy Planning Station version 5.1.1 (Accuray). The field width and pitch used were 1.05‐cm and 0.127. The prescription dose was calculated using the convolutional/superposition algorithm with the fine dose grid. All HT plans were delivered by 6 MV FFF x‐ray beams of TomoHD (Accuray). Since the TomoTherapy Planning Station lacks a scaling function, optimization was performed to achieve a prescription dose exceeding D99.5% but as close as possible to it (i.e., D99.5% ≤ prescription dose < D100%).

### 2.6 CK

For CK, treatment planning was performed by CK MultiPlan TPS version 3.2.0 (Accuray). CK G4 with Fixed and Iris collimator was used as the treatment machine. The collimators selected for use were a combination of those not exceeding the maximum diameter of the tumor and those smaller than it. The collimator sizes used were 1–3 selected from 5, 7.5, and 10 mm for the SRS group and 3–5 selected from 7.5, 10, 12.5, 15, 20, and 30 mm for the SRT group. To adjust the dose distribution for all cases, three shells, taking into account collimator size, were created outside the PTV and planned using a sequential optimization. The prescription dose was calculated using the Monte Carlo algorithm with a 2% uncertainty on a 1 × 1 × 1.25‐mm resolution. All CK plans were delivered by 6 MV x‐ray beams of CK with Iris collimator.

### 2.7 GK

For GK, the treatment plan was generated using the Leksell GammaPlan TPS with the fast inverse planning dose optimizer, commercially referred to as Lightning version 11.3.2 (Elekta). The GK Icon system used in this study consists of 8 movable sectors with a total of 192^60^Co sources, where the sectors can be set to three different collimator sizes (4, 8, and 16 mm in diameter) and beam blocking positions to match closely the treatment volume to the intended target volume. Treatment time (t‐time) was calculated at a dose rate of 2.365 Gy/min. The prescription dose was calculated using the TMR10 algorithm with a resolution of 1 × 1 × 1‐mm.

### 2.8 ZAP‐X

For ZAP‐X, treatment planning was performed by ZAP‐X TPS version 1.8.58.12369 (Zap Surgical Systems). The multiple isocenters and collimators were selected to align with the shape and size of the PTV. For some cases, forward planning as well as GK was used to shape the 50% isodose line according to the shape of the tumor, while inverse planning was used to calculate the other cases. The collimator sizes used were 1–3 selected from 4, 5, 7.5, 12.5, and 15 mm for the SRS group and 3–5 selected from 7.5, 10, 12.5, 15, 20, and 25 mm for the SRT group. In other cases, inverse planning was performed after forward planning. This involves conducting forward planning with the isocenter placement intended by the physician to achieve a rough coverage initially, followed by inverse planning to provide more conformal irradiation. The prescription dose was calculated using the Ray Tracing algorithm on a 1 × 1 × 1‐mm resolution. All ZAP‐X plans were delivered by 3 MV x‐ray beams.

### 2.9 Dosimetry and irradiation parameters

Dosimetry used for comparison included: Gradient Index (GI), Paddick Conformity Index (CI), V12 Gy and V24Gy. Irradiation parameter was the t‐time. GI is a measure of low dose spread to surrounding normal tissue outside the target volume.^11^ GI was calculated using the following formula:

$$GI\;=\frac{PIV_{50\%}}{PIV}$$

where PIV_50%_ was 50% of the prescription isodose line volume, PIV was the volume covered by the prescription isodose line. CI was calculated using the following formula:

$$CI=\frac{TV_{PIV}}{TV}\;\times \frac{TV_{PIV}}{PIV}$$

where TV*_PIV_* was the volume of the target covered by the prescription isodose line, TV was the target volume. Radiation brain necrosis (RBN) is a complication that occurs after SRS and SRT.^12^ Previous studies have associated the risk of RBN with V12 Gy for SRS and V24 Gy for SRT.^13^, ^14^, ^15^ Therefore, the SRS and SRT groups were evaluated at V12 Gy and V24 Gy, respectively. The t‐time was calculated using the TPS and represents the recorded duration, excluding the setup time if it was included.

### 2.10 Statistical analysis

All Plan dose measurements were performed using MIM Maestro ver.7.2.9 (MIM Software, Inc., Cleveland, OH, USA). Statistical analysis was performed using RStudio (RStudio: Integrated Development for R. RStudio, PBC, Boston, MA, USA). A one‐way analysis of variance (ANOVA) was performed to test for statistical significance among the six treatment techniques. The post‐hoc tests were Dunnett's test between ZAP‐X and the other treatment techniques. A p‐value of < 0.05 was considered indicative of statistical significance.

## 3 RESULTS

**FIGURE 1:**
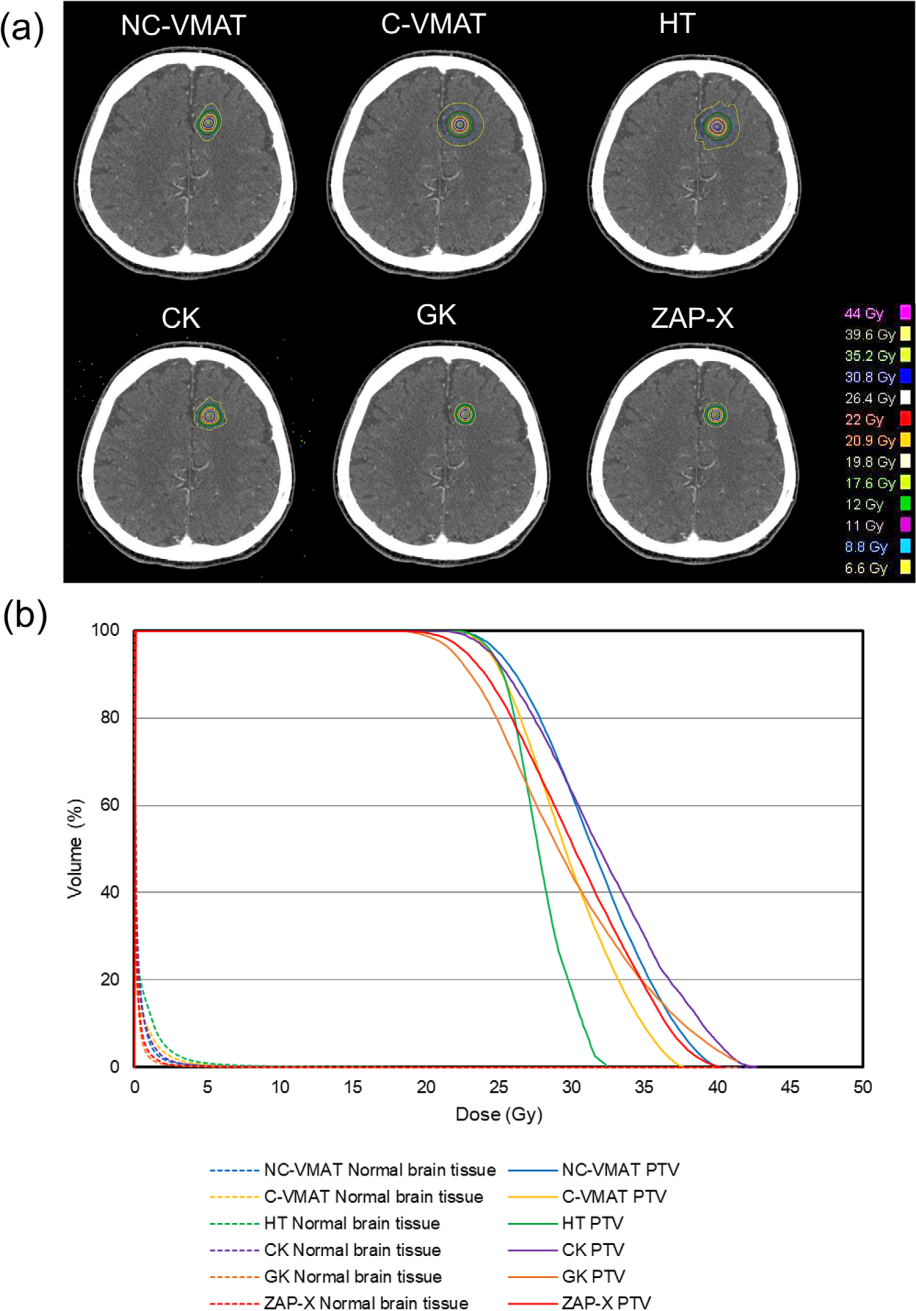
The dose distribution (a) and DVH (b) for representative cases (patient 3: PTV 0.3 cc, Prescription dose 22 Gy) of each of the six planning techniques in the SRS group. (green line: Region surrounded by a dose of 12 Gy) ＊CK utilizes the Monte Carlo algorithm, resulting in dose distribution plots in the air. DVH, dose‐volume histogram.

**FIGURE 2:**
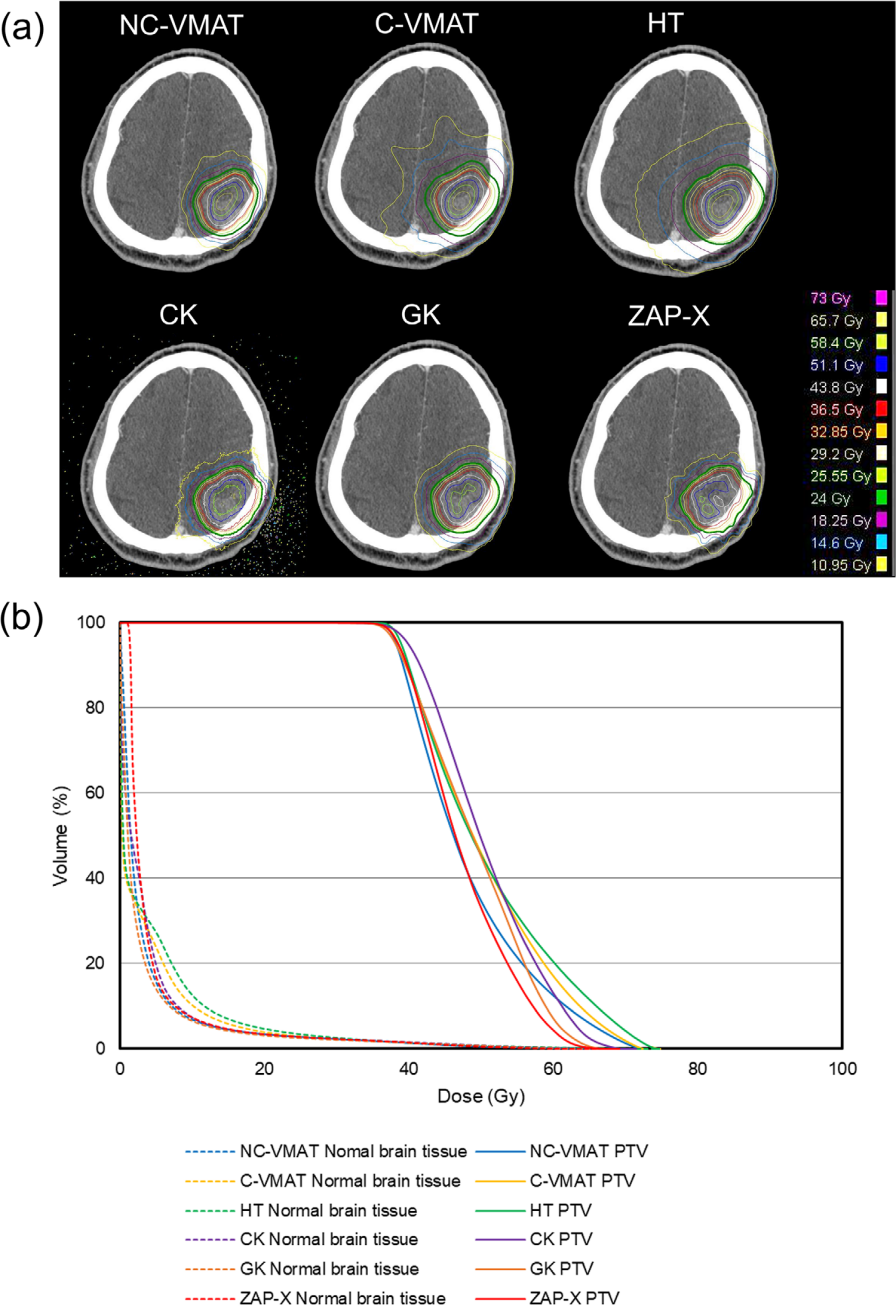
The dose distribution (a) and DVH (b) for representative cases (patient 9: PTV 24.0 cc, Prescription dose 36.5 Gy) of each of the six planning techniques in the SRT group. (green line: Region surrounded by a dose of 24  Gy) ＊CK utilizes the Monte Carlo algorithm, resulting in dose distribution plots in the air. DVH, dose‐volume histogram.

The median PTV of the SRS and SRT groups was 0.4 cc (range 0.3–1.0) and 12.2 cc (range 2.4–26.9), respectively. Figure 1 shows (a) the dose distribution and (b) dose‐volume histogram (DVH) for representative cases (patient 3: PTV 0.3 cc, Prescription dose 22 Gy) of each of the six planning techniques in the SRS group. The NC‐VMAT, CK, GK and ZAP‐X have steeper dose distributions than the C‐VMAT and HT. The V12 Gy in this case was 1.91, 2.35, 2.65, 1.32, 0.74, and 0.88 cc for NC‐VMAT, C‐VMAT, HT, CK, GK, and ZAP‐X, respectively. Regarding DVH, the PTV maximum dose of HT was smaller than that of other techniques. Figure 2 shows (a) the dose distribution and (b) DVH for representative cases (patient 9: PTV 24.0 cc, Prescription dose 36.5 Gy) of each of the six planning techniques in the SRT group. Similar to the cases in the SRS group, the NC‐VMAT, CK, GK, and ZAP‐X have steeper dose distributions than the C‐VMAT and HT. The V24 Gy in this case was 42.60, 46.01, 52.26, 41.18, 37.91, and 42.03 cc for NC‐VMAT, C‐VMAT, HT, CK, GK, and ZAP‐X, respectively. Regarding DVH, the PTV maximum dose was approximately the same for all techniques. Table 2 shows the mean values and standard deviations (SD) of the dosimetric parameters for each treatment plan in the SRS and SRT groups. For the SRS group, the mean values of GI were lowest in the following order: GK, ZAP‐X, NC‐VMAT, CK, HT, and C‐VMAT. Comparing ZAP‐X as a reference, no significant difference was observed only for GK (*p* = 0.995). The mean values of CI were highest in the following order: GK, ZAP‐X, CK, NC‐VMAT, C‐VMAT, and HT. Comparing ZAP‐X as a reference, no significant difference was observed only for GK (*p* = 0.998). The mean values of V12 Gy were lowest in the following order: GK, ZAP‐X, CK, NC‐VMAT, HT, and C‐VMAT. Comparing ZAP‐X as a reference, significant differences were observed for C‐VMAT and HT (*p* < 0.05). For the SRT group, the mean values of GI were lowest in the following order: GK, ZAP‐X, CK, NC‐VMAT, HT, and C‐VMAT. Comparing ZAP‐X as a reference, significant differences were observed for C‐VMAT and HT (*p* < 0.05). The mean values of CI were highest in the following order: GK, ZAP‐X, NC‐VMAT, C‐VMAT, CK and HT. Comparing ZAP‐X as a reference, significant difference was observed only for HT (*p* < 0.05). The mean values of V24 Gy were lowest in the following order: GK, CK, ZAP‐X, NC‐VMAT, C‐VMAT, and HT. Comparing ZAP‐X as a reference, no significant differences were observed for all treatment techniques. To enhance reliability given the large variability, the data from patient 8 and 9, which had larger tumor volumes, were excluded. The mean values and SD for the remaining four cases were analyzed. The mean values of V24 Gy for the four cases were lowest in the following order: GK, ZAP‐X, CK, NC‐VMAT, C‐VMAT, and HT. Comparing ZAP‐X as a reference, no significant differences were observed for all treatment techniques. Table 3 shows the mean values and SD of t‐time for each treatment plan in the SRS and SRT group. For the SRS group, the mean values of t‐time were shortest in the following order: C‐VMAT, NC‐VMAT, HT, ZAP‐X, CK and GK. Comparing ZAP‐X as a reference, no significant difference was observed only for CK (*p* = 0.990). For the SRT group, the mean values of t‐time were shortest in the following order: C‐VMAT, NC‐VMAT, HT, ZAP‐X, GK and CK. Comparing ZAP‐X as a reference, no significant differences were observed for CK (*p* = 0.160) and GK (*p* = 0.560).

**TABLE 2:** The dosimetry of stereotactic radiosurgery and stereotactic radiotherapy groups.

Parameter	ZAP‐X mean ± SD	NC‐VMAT mean ± SD	*p*‐Value	C‐VMAT mean ± SD	*p*‐Value	HT mean ± SD	*p*‐Value	CK mean ± SD	*p*‐Value	GK mean ± SD	*p*‐Value
SRS group											
GI (*n* = 6)	2.99 ± 0.16	4.28 ± 0.80	0.042^*^	5.61 ± 1.44	< 0.001^*^	4.68 ± 0.89	0.008^*^	4.31 ± 1.05	0.048^*^	2.81 ± 0.14	0.995
CI (*n* = 6)	0.80 ± 0.03	0.60 ± 0.10	< 0.001^*^	0.44 ± 0.12	< 0.001^*^	0.42 ± 0.11	< 0.001^*^	0.61 ± 0.15	0.007^*^	0.82 ± 0.07	0.998
V12 Gy (cc) (*n* = 6)	1.58 ± 0.72	2.87 ± 1.35	0.523	5.31 ± 3.35	0.002^*^	4.23 ± 1.08	0.034^*^	2.32 ± 0.86	0.999	1.40 ± 0.78	0.999
SRT group											
GI (*n* = 6)	2.80 ± 0.15	3.27 ± 1.01	0.417	3.81 ± 0.50	0.010^*^	3.76 ± 0.35	0.014^*^	2.98 ± 0.49	0.962	2.61 ± 0.03	0.953
CI (*n* = 6)	0.84 ± 0.03	0.84 ± 0.07	0.999	0.82 ± 0.08	0.980	0.65 ± 0.13	< 0.001^*^	0.77 ± 0.09	0.360	0.90 ± 0.04	0.550
V24 Gy (cc) (*n* = 6)	22.27 ± 19.12	22.45 ± 16.44	0.999	23.74 ± 17.93	0.999	31.62 ± 26.64	0.860	21.99 ± 17.05	0.999	19.30 ± 15.86	0.999
V24 Gy (cc) (*n* = 4, except for patients 8 and 9)	10.23 ± 4.25	12.11 ± 4.58	0.973	12.47 ± 4.99	0.946	15.41 ± 6.39	0.447	11.26 ± 4.43	0.998	9.29 ± 4.16	0.998

Abbreviations: CI, Paddick Conformity Index; CK, CyberKnife; C‐VMAT, coplanar VMAT; GI, Gradient Index, GK, Gamma Knife; HT, Helical TomoTherapy; NC‐VMAT, non‐coplanar volumetric modulated arc radiotherapy; SD, standard deviation; SRS, stereotactic radiosurgery; SRT, stereotactic radiotherapy; VxxGy, volume to xxGy of normal tissue brain.

* *p* < 0.05.

**TABLE 3:** Evaluation of treatment time for stereotactic radiosurgery and stereotactic radiotherapy groups.

Parameter	ZAP‐X mean ± SD	NC‐VMAT mean ± SD	*p*‐Value	C‐VMAT mean ± SD	*p*‐Value	HT mean ± SD	*p*‐Value	CK mean ± SD	*p*‐Value	GK mean ± SD	*p*‐Value
SRS group (*n* = 6)											
Treatment time (min)	26.47 ± 11.50	7.20 ± 1.24	< 0.001^＊^	4.89 ± 1.73	< 0.001^＊^	18.22 ± 5.35	0.044^＊^	28.17 ± 6.77	0.990	35.43 ± 11.39	0.027^＊^
SRT group (*n* = 6)											
Treatment time (min)	24.72 ± 5.55	4.24 ± 1.00	< 0.001^＊^	1.89 ± 0.55	< 0.001^＊^	14.05 ± 3.78	< 0.001^＊^	29.83 ± 3.43	0.160	27.93 ± 7.03	0.560

Abbreviations: CK, CyberKnife; C‐VMAT, coplanar VMAT; GK, Gamma Knife; HT, Helical TomoTherapy; NC‐VMAT, non‐coplanar volumetric modulated arc radiotherapy; SD, standard deviation; SRS, stereotactic radiosurgery; SRT, stereotactic radiotherapy.

* *p* < 0.05.

## 4 DISCUSSION

The study investigated various treatment techniques in radiation therapy for BT, comparing dosimetry and delivery parameters in the SRS and SRT groups. The techniques studied included NC‐VMAT, C‐VMAT, HT, CK, GK, and the new device ZAP‐X. In this study, V12 and V24 Gy were evaluated as dose indicators for the normal brain. Previous studies have indicated an increased risk of RBN at V12 and V24 Gy doses higher than 10.0 and 16.8 cc, respectively.^15^, ^16^ In the SRS group, the mean V12 Gy was consistently below 10.0 cc for all treatment techniques, while in the SRT group, the mean V24 Gy was higher than 16.8 cc. Due to the large SD of V24 Gy across all techniques, patients with larger tumor volumes were excluded to calculate and evaluate the mean values. The mean V24 Gy for the remaining four cases was 16.8 cc or less. V12 and V24 Gy are affected by prescription dose, tumor size, and tumor shape.^15^ Therefore, the reason for this may be that the study was evaluated using prescription doses used in actual clinical practice, including cases with high prescription doses. Furthermore, we consider that this is due to the inclusion of cases with large tumor volume. The mean values of GI, CI, and V12 Gy for the SRS group were better for GK than for the other treatment techniques. Regarding GI and CI, the results were similar to those reported by Paddick et al.^9^ Moreover, the mean values of GI, CI, and V24 Gy for the SRT group were also better for GK than for the other treatment techniques. Wang et al.^7^ conducted a comparative study of dose characteristics in cases of a single BT using CK, GK, and ZAP‐X. The study employed different normalization methods for GK and the other techniques; they reported that the CI for GK was significantly lower than for ZAP‐X and CK, while the GI was not significantly different between the techniques. Although CK and GK were not directly compared in this study, CK and GK results in the SRT group were not significantly different from ZAP‐X, a trend similar to previous study. The dose measurement results of NC‐VMAT, C‐VMAT, and HT improved for the SRT group compared to the SRS group. In particular, NC‐VMAT was close to the mean values observed for ZAP‐X, CK, and GK. Kim et al.^17^ reported that the CI of VMAT was superior to that of GK when the tumor volume of vestibular schwannoma (VS) was greater than 0.5 cm^3^. Moreover, Dong et al.^18^ compared GK, CK, and VMAT for BTs > 3 cm; GI, CI, and t‐time values were similar in this study. Regarding the evaluation based on target volume, due to the limited number of cases in this study, further study is necessary. However, these findings suggest that the computational results of NC‐VMAT, C‐VMAT, and HT improve with an increase in tumor volume. Furthermore, the better GI of ZAP‐X, as well as GK, in both groups may be attributed to the fact that ZAP‐X utilizes energy as low as 3 MV, which reduces the scattered penumbra. Additionally, the short SAD for ZAP‐X, at 450‐mm, contributes to a decrease in geometric penumbra.^9^ GI and V12 Gy of NC‐VMAT in the SRS group and GI and V24 Gy in the SRT group were smaller than those of C‐VMAT. This result is similar to that reported by Zhang et al.^19^ and shows the dose attenuation advantage of non‐coplanar irradiation. Furthermore, in their study, C‐VMAT was performed with 2 arcs, while in this study, it was performed with 1 arc. These results suggest that increasing the number of arcs in C‐VMAT does not improve the distribution. In the SRS group, the maximum dose of PTV was lower for HT than for other treatment techniques, especially in cases with small PTV. The reason for this was that the field width was 1.05‐cm, which did not allow for a higher central dose. The mean value of CI was the smallest for HT, as HT delivers the dose with a helical parallel fan beam, whereas ZAP‐X, NC‐VMAT, CK, and GK use non‐isocentric, non‐coplanar beams from different angles.^20^ Moreover, the SRT group had the largest mean V24 Gy. This result was similar to the finding reported by Agostinelli et al.^21^ that normal brain dose increases as target volume increases. The mean value of t‐time for ZAP‐X, CK and GK were longer than those for other treatment techniques. NC‐VMAT, C‐VMAT, and HT are advantageous when treating patients who have difficulty remaining in the supine position for extended periods of time. These results were similar to those of previous studies.^9^, ^20^, ^22^, ^23^ The longer mean GK t‐time was likely due to the maximum diameter of the collimator (16 mm), necessitating multiple shots and a lower dose rate. In this study, the dose rate was 2.365 Gy/min, but using new sources could reduce the time.^24^ ZAP‐X and CK are likely affected by the time required for kV imaging for position correction.^25^ Furthermore, ZAP‐X moves on two rotational axes, so translational repositioning of the beam is not possible. Therefore, the patient couch must be moved to obtain a new translational position of the beam relative to the patient. This is another factor that increases the t‐time required.^26^ For these reasons, it is important to select the treatment based on the tumor volume and the patient condition.

This study has several limitations. First, the prescription isodose volume and PTV maximum dose were aligned as closely as possible to GK and ZAP‐X in this study to allow for comparative evaluation under aligned conditions. However, when dealing with NC‐VMAT, C‐VMAT, HT, and CK, it is common to adopt a different approach when creating plans. Furthermore, differences in the prescription isodose line change the dosimetry parameter evaluated in this study.^27^, ^28^ Second, it is also worth noting that while CK MultiPlan TPS was utilized for CK planning in this study, the current CK planning system has migrated to Precision TPS (Accuray). This replacement creates the potential for variability in CK results due to the use of the new VOLO algorithm. Schüler et al.^29^ reported no significant change in CI, GI when using VOLO optimization compared to sequential optimization. However, there were significant improvements in the number of MU, number of beams, and t‐time. Moreover, Thiele et al.^30^ reported that in CK treatment for VS, VOLO demonstrated significantly lower maximum doses to OAR compared to sequential optimization, with a statistically significant difference observed. These reports suggest that the planning approach with VOLO may affect the results of CK in this study. Third, this study was evaluated with six patients in each of the two groups. However, increasing the sample size could potentially alter the results. Additionally, the substantial variability in volume observed, particularly in the SRT group, may influence the outcomes. These issues need to be further investigation in future study. Forth, in this study, the MLC size used for NC‐VMAT and C‐VMAT was leaves of 5‐mm. The use of equipment with smaller micro‐MLC could potentially alter the results. In particular, CI has been reported to be improved when micro‐MLC is used than when MLC leaves of 5‐mm is used.^31^, ^32^ Fifth, although there were no cases in close proximity to OAR in this study, future study should evaluate the characteristics of each technique in cases in such cases. Finally, dose painting was not evaluated in this study.^33^ It should be evaluated in future study.

## 5 CONCLUSION

ZAP‐X demonstrates relatively superior CI and GI for small tumors, similar to GK. However, differences between ZAP‐X, NC‐VMAT, CK, and GK diminish as tumor volume increases. The t‐time for NC‐VMAT, C‐VMAT, and HT is shorter compared to ZAP‐X, CK, and GK, irrespective of tumor volume. Based on the results of this study, if time efficiency is the primary consideration, VMAT with its shorter t‐time is more suitable for treating a single BT. The data obtained from this study will be crucial and beneficial in the decision‐making process for selecting radiation therapy for BT.

## AUTHOR CONTRIBUTIONS

*Study conception and design*: Toshihiro Suzuki, Masahide Saito, Hiroshi Onishi, Ryutaro Nomura, and Hiroshi Takahashi. *Acquisition of data*: Toshihiro Suzuki, Masahide Saito, Ryutaro Nomura, Hikaru Nemoto, Naoto Yanagisawa, and Ryuma Sawada. *Analysis and interpretation of data*: Toshihiro Suzuki, Masahide Saito, Ryutaro Nomura, Hikaru Nemoto, and Ryuma Sawada. *Drafting of manuscript*: Toshihiro Suzuki, Masahide Saito, Hiroshi Onishi, Toshihiro Suzuki, Masahide Saito, Ryutaro Nomura, Hikaru Nemoto, Ryuma Sawada, Zennosuke Mochizuki, Naoki Sano, and Hiroshi Takahashi. *Critical revision*: Toshihiro Suzuki, Masahide Saito, Hiroshi Onishi, and Hiroshi Takahashi.

## CONFLICT OF INTEREST STATEMENT

There are no conflicts of interest to declare.

## ETHICS STATEMENT

This study was approved by the institutional review board (IRB) of Kasugai General Rehabilitation Hospital (receipt number: 2023–4).

## Data Availability

All data generated and analyzed during this study are included in this published article (and its supplementary information files).
